# Prevalence of Same-Sex Sexual Behavior and Associated Characteristics among Low-Income Urban Males in Peru

**DOI:** 10.1371/journal.pone.0000778

**Published:** 2007-08-22

**Authors:** Jesse L. Clark, Carlos F. Caceres, Andres G. Lescano, Kelika A. Konda, Segundo R. Leon, Franca R. Jones, Susan M. Kegeles, Jeffrey D. Klausner, Thomas J. Coates, NIMH STD/HIV Collaborative Prevention Trial

**Affiliations:** 1 Division of Infectious Diseases, David Geffen School of Medicine, University of California at Los Angeles, Los Angeles, California, United States of America; 2 Unidad de Salud, Sexualidad y Desarrollo Humano, Universidad Peruana Cayetano Heredia, Lima, Peru; 3 United States Naval Medical Center Research Detachment, Lima, Peru; 4 Naval Medical Research Center, Biological Defense Research Directorate, Silver Spring, Maryland, United States of America; 5 Center for AIDS Prevention Studies, University of California at San Francisco, San Francisco, California, United States of America; 6 San Francisco Department of Public Health, San Francisco, California, United States of America; 7 The National Institute of Mental Health Multisite International Group, Bethesda, Maryland, United States of America; School of Public Health and Family Medicine, South Africa

## Abstract

**Background:**

Peru has a concentrated HIV epidemic in which men who have sex with men are particularly vulnerable. We describe the lifetime prevalence of same-sex sexual contact and associated risk behaviors of men in Peru's general population, regardless of their sexual identity.

**Methods and Results:**

A probability sample of males from low-income households in three Peruvian cities completed an epidemiologic survey addressing their sexual risk behavior, including sex with other men. Serum was tested for HSV-2, HIV, and syphilis. Urine was tested for chlamydia and gonorrhea. A total of 2,271 18–30 year old men and women were contacted, of whom 1,645 (72.4%) agreed to participate in the study. Among the sexually experienced men surveyed, 15.2% (85/558, 95% CI: 12.2%–18.2%) reported a history of sex with other men. Men ever reporting sex with men (MESM) had a lower educational level, had greater numbers of sex partners, and were more likely to engage in risk behaviors including unprotected sex with casual partners, paying for or providing compensated sex, and using illegal drugs. MESM were also more likely to have had previous STI symptoms or a prior STI diagnosis, and had a greater prevalence of HSV-2 seropositivity.

**Conclusions:**

Many low-income Peruvian men have engaged in same-sex sexual contact and maintain greater behavioral and biological risk factors for HIV/STI transmission than non-MESM. Improved surveillance strategies for HIV and STIs among MESM are necessary to better understand the epidemiology of HIV in Latin America and to prevent its further spread.

## Introduction

HIV infection in Peru is concentrated within the core risk group of men who have sex with men (MSM). Surveillance studies of HIV infection in Peru have noted a prevalence in MSM ranging from 11.0–18.5%, but only 1.6% in female sex workers (FSWs) and less than 1% in the general population [1]–[5]. Previous studies have identified several behavioral and biological factors associated with the higher prevalence of HIV among MSM [5]–[10]. However, current understandings of HIV epidemiology among Peruvian MSM are based on studies with venue-based convenience samples that do not provide a complete understanding of the lifetime risk factors or an accurate estimate of the prevalence of HIV and sexually transmitted infections (STIs) among *all* men who have engaged in sex with men.

The diversity of sexual identities associated with male homosexual behavior in Latin America has been analyzed in a number of studies [5], [11]–[19]. Researchers studying same-sex sexual contact and related risk behaviors among Latino men have described a construction of sexuality that links penetration with masculinity and receptive intercourse with femininity, through which male same-sex sexual contact does not necessarily presume a homosexual identity [19]–[27]. However, this analysis of male-male sexuality is based on studies of Latino communities in the United States, who have been partially or thoroughly influenced by U.S. gay communities and cultures. Understanding of male same-sex contact and associated risk behaviors within Latin America itself is an important area that has only begun to be explored [5], [6], [13], [18], [28], [29].

Previously published estimates of STI and HIV prevalence among MSM in Peru have found high levels of HIV and syphilis infection [5], [9], [10]. However, these estimates were based on samples of men attending MSM venues and STI clinics, or identified as MSM by peer recruiters. A recent analysis of sampling methodologies in the U.K. suggests that community or venue-based sampling not only overrepresents the highest risk segment of the population, but also excludes the potentially large number of men who have sex with men but exist outside of these community frameworks [30]. Particularly when addressing male same-sex behavior in Latin America, failure to include men who do not identify with established GBT social settings and geographic locales provides a skewed understanding of risk behavior and disease prevalence, and neglects the men most likely to serve as a “bridge” between core risk groups and the general population.

We conducted a random household survey of adults in three coastal cities in Peru to assess prevalence and risk factors for HIV and STI transmission in low-income populations. As a secondary analysis, we examined same-sex contact among men in the study population, all of whom were recruited outside of MSM venues or social circles and regardless of their identification with male homosexuality or contemporary participation in an MSM network. We sought to provide a comprehensive description of the characteristics of men who have ever had sex with men (MESM) in Peru's general population and to study the association of MESM activity with sexual risk behavior and risk for STIs.

## Methods

### Study Design, Population and Recruitment

We conducted a cross-sectional study of sexual activity, risk factors for HIV/STI transmission, and prevalence of HIV/STIs among a population-based sample of men recruited from 2000–2001 in three coastal cities in Peru. The coastal region maintains the greatest density of urban areas in Peru, whose low-income *barrios* (neighborhoods) maintain HIV prevalences considered to be among the highest in the country.

During the exploratory phase of the NIMH Collaborative HIV/STD Prevention Trial, 34 *barrios* in Lima, Trujillo, and Chiclayo were defined as low-income areas, according to the Unmet Basic Needs Index [31]. The total population of the *distritos* (districts) studied ranged from 20,000–260,000, within which each of the *barrios* contained approximately 250–350 households, with an average of 3.7 residents per household [32]. A detailed household map of each *barrio* was prepared from census data and ethnographic reports, and 75 households within each barrio were selected at random. All individuals in a selected household were enumerated by study staff, and one eligible individual from each household was identified at random for recruitment. If the selected individual was not available at the time of the initial visit, study staff made up to two additional visits to the household in an effort to contact them. Potential recruits who could not be contacted after a maximum of three visits were excluded from the study. Demographic data were not recorded for potential recruits who could not be contacted or who declined participation before providing informed consent. Individuals between 18–30 years old who planned to remain in the *barrio* for at least two years were eligible. All respondents agreeing to participate provided written informed consent. The study protocol was approved by the Committee of Human Subjects Research of the University of California at Los Angeles, the University of California at San Francisco, the Universidad Peruana Cayetano Heredia, and the U.S. Naval Medical Research Center in Bethesda, MD in compliance with all federal regulations regarding the protection of human subjects.

### Data Collection

During the informed consent process, participants were informed that they would be asked questions concerning their risks for HIV and STIs, including sexual behavior, though same-sex behavior was not explicitly addressed. Participants completed the survey in a temporary project office located in their *barrio* with the use of an ACASI (Audio Computer-Assisted Self-Interviewing) system. Individuals responded to questions in Spanish about their socio-demographic background, sexual behavior, risk factors for HIV and STIs, and substance use. Participants completed the interview independently in a private room, though staff members were available to answer any questions. Data were collected anonymously and participants were assured of the confidentiality of their responses prior to completing the questionnaire. Study staff collected blood and urine samples from participants and provided pre-test counseling. Approximately one month following the initial evaluation, participants returned for a follow-up visit where they received post-test counseling and the results of their screening tests. Anyone diagnosed with an STI was given appropriate antibiotic therapy or, in the case of HIV infection, referral to a Ministry of Health facility for ongoing care. No follow-up data were collected as part of this exploratory survey.

### Laboratory Methods

All blood and urine samples were analyzed at the U.S. Naval Medical Research Center Detachment in Lima, Peru. Blood was screened for syphilis infection by RPR assay (RPRnosticon, Biomérieux; Marcy l'Étoile, France) with Treponema Pallidum Particle Agglutination (TPPA) confirmation of positive results (Serodia, Fujirebio; Tokyo, Japan). Two separate ELISA assays were used to diagnose HIV-1 (Vironostika, Biomérieux; Marcy l'Étoile, France; Genetic Systems, Biorad; Hercules, CA). HIV-positive samples were analyzed by Western Blot to confirm infection (Genetic Systems, Biorad; Hercules, CA). HSV-2 specific ELISA (HerpeSelect, Focus Technologies; Cypress, CA) was used for serologic detection of genital herpes, with seropositivity defined by a minimum ratio of 1.10. Urine specimens were analyzed with nucleic acid amplification testing (Roche Amplicor, Roche Diagnostics; Alameda, CA) for the presence of urethral gonorrhea or chlamydia.

### Data Analysis

MESM were defined by participants' response to the question ‘Have you ever had sex with a man?’ and analyzed as a dichotomous (Yes/No) variable. As no definition of “sex” was given in the survey, the exact meaning of “sex” was participant-defined. Association with sexual risk behavior, socio-demographic variables, and STI prevalence was analyzed with contingency tables and Chi-square tests. For the lifetime total number of sex partners, a binary logarithm was used to accommodate for the highly skewed distribution of this variable. Individuals with missing data were excluded from the affected analysis only. Multivariate regression used stepwise comparison to determine the order of variables entering the model and used likelihood-ratio tests to determine the statistical significance of each variable. All confidence intervals were calculated at 95%. Stata 9.1 software was used for all analyses (Stata Corporation, College Station, TX).

## Results

### Sociodemographic Factors

A total of 2,550 men and women were targeted for recruitment, of whom 2,271 were contacted and 1,645 (72.4%) were eligible and agreed to complete the survey. Almost all participants (1,635/1,645; 99.4%) provided biological samples. There were no significant differences in refusal rates or in behavioral/biological findings between the study sites. A total of 654 men were included in the study, though 96 (14.7%) denied any previous sexual contact and were excluded from further analysis. Any lifetime same-sex sexual contact was reported by 15.2% of all sexually active men (85/558, 95% CI: 12.2%–18.2%), and by 15.9% of sexually active eighteen year olds (11/69, 95% CI: 8.2%–26.7%), with no age-related differences in MESM prevalence observed (Table 1). MESM were generally less educated, with 64.7% having graduated from high school, as opposed to 81.2% of non-MESM (p = 0.001). MESM were also more likely than exclusively heterosexual men to be married or divorced (32.9% vs. 24.3%; p = 0.040), rather than single.

**Table 1:** Participant characteristics and risk behaviors among general population males: Lima, Chiclayo, and Trujillo; Peru, 2001.

Variables†	Non-MESM	MESM	p-value‡
	n = 473	n = 85	
	n (%)	n (%)	
**Socio-demographic characteristics**			
Age, years (mean±standard deviation)	22.6±3.54	22.9±3.62	0.385
Graduated high school	384 (81.2%)	55 (64.7%)	0.001
Marital Status			
Single	358 (75.7%)	57 (67.1%)	0.040
Married	101 (21.3%)	21 (24.7%)	
Divorced/Widowed	14 (3.0%)	7 (8.2%)	
**Risk Behaviors**			
No. of sex partners (mean±standard deviation)			
Lifetime	4.5±7.06	10.1±14.9	0.001
12 Months	1.6±2.95	2.7±3.67	0.010
3 Months	0.8±1.07	1.3±2.64	0.074
Had unprotected sex with a casual partner(s), 3 months*	18 (4.0%)	8 (9.6%)	0.026
Had unprotected sex with steady partner, 3 months	142 (33.1%)	30 (39.0%)	0.317
Paid for sex, 12 months	49 (11.1%)	17 (20.7%)	0.016
Received compensation for sex, Lifetime	54 (11.5%)	31 (36.5%)	<0.001
Frequency of alcohol use			
Daily or weekly	118 (25.0%)	27 (31.7%)	0.344
Monthly	218 (46.2%)	31 (36.5%)	
Yearly	83 (17.6%)	18 (21.2%)	
Never	53 (11.2%)	9 (10.6%)	
Used illegal drugs**, last 3 months	33 (7.0%)	15 (17.7%)	0.001
Know someone with HIV-infection	92 (19.7%)	26 (31.0%)	0.021
Ever tested for HIV	80 (31.9%)	60 (40.0%)	0.674

† Totals may vary as some variables have missing data

‡ p-values were calculated from Chi-square tests except for age and number of sex partners, which were calculated with Student t-tests

* Gender of casual and steady partners was not recorded

** Marijuana or cocaine

### Risk Behavior

MESM had a greater number of sex partners than non-MESM, both in their lifetime (10.1 vs. 4.5 partners; p = 0.001) and in the past year (2.7 vs. 1.6; p = 0.010), and more often reported unprotected intercourse with a casual partner in the past 3 months (9.6% vs. 4.0%; p = 0.026), though the gender of these partners was not specified. Additionally, MESM were more likely than men who did not report same-sex contact to have engaged in commercial sex transactions, either paying another person for sex within the previous year (20.7% vs. 11.1%; p<0.001), or having been paid for sex in their lifetime (36.5% vs. 11.5%; p<0.001).

When asked to evaluate their personal risk for HIV, MESM were more likely to rate themselves as being at “moderate” or “high” risk for infection (30.8% vs. 10.5%; p<0.001), and to know someone who was HIV-positive (31.0% vs. 19.7%, p = 0.021). Despite their greater perception of personal risk, MESM were no more likely to have attempted to obtain condoms in the past three months, though the majority of men who sought condoms (143/182; 78.6%) stated that it was “easy” to get them. MESM were also no more likely than exclusively heterosexual men to have been tested for HIV infection in the past.

### Prevalence of STIs and STI Symptoms

HSV-2 seropositivity was dramatically higher in MESM than in men who did not report same-sex contact (21.4% vs 5.3%; p<0.001; Table 2). The prevalence of syphilis was also higher among MESM (2.4% vs. 0.7%; p = 0.126), and the prevalence of chlamydia was slightly higher (6.0% vs. 4.5%; p = 0.633), though neither difference reached statistical significance. No cases of HIV (0%, 95% CI: ≤0.002%) or urethral gonorrhea (0%, 95% CI: ≤0.002%) were diagnosed in any male study participant. MESM were more likely than non-MESM to report a previous genital ulcer (20.2% vs. 10.9%; p = 0.016), genital discharge (21.4% vs. 7.9%; p<0.001), or an STI diagnosis (26.0% vs. 6.7%; p <0.001).

**Table 2:** Prevalence and history of sexually transmitted infections (STIs) among general population males: Lima, Chiclayo, and Trujillo; Peru, 2001.

	Non-MESM	MESM	p-value
	(N = 473)	(N = 85)	
	n (%)	n (%)	
**STI history**			
History of STI Diagnosis	32 (6.7%)	22 (26.0%)	<0.001
History of Genital Ulcer	51 (10.9%)	17 (20.2%)	0.016
History of Genital Discharge	37 (7.9%)	18 (21.4%)	<0.001
**STI Prevalence**			
Gonorrhea (Urethral)	0 (0%)	0 (0%)	N/A
Chlamydia (Urethral)	21 (4.5%)	5 (6.0%)	0.633
HSV-2	25 (5.3%)	18 (21.4%)	<0.001
Syphilis	3 (0.7%)	2 (2.4%)	0.126
HIV	0 (0%)	0 (0%)	N/A

### Multivariate Analysis

Lower educational level, increased number of sex partners, provision of compensated sex, history of an STI, and HSV-2 seropositivity all remained associated with same-sex sexual contact after multivariate adjustment (Table 3).

**Table 3:** Multivariate analysis of socio-demographic and risk behavior characteristics associated with MESM behavior among general population men: Lima, Chiclayo, and Trujillo; Peru, 2001.

Variable	Unadjusted OR	Adjusted OR**	95% CI	p-value
Graduated high school				
No	Ref	Ref	-	-
Yes	2.35 (0.001)	2.01	1.10–3.65	0.022
Marital Status				
Single or currently married	Ref	Ref	-	-
Previously married	2.94 (0.024)	2.93	0.96–8.96	0.058
Total number of sex partners, lifetime*	1.77 (<0.001)	1.42	1.13–1.79	0.003
Compensated for sex, lifetime				
No	Ref	Ref	-	-
Yes	4.41 (<0.001)	2.51	1.29–4.90	0.007
Illegal drug use, 3 months				
No	Ref	Ref	-	-
Yes	2.83 (0.002)	0.90	0.36–2.23	0.822
HSV-2 Seropositive				
No	Ref	Ref	-	-
Yes	4.83 (<0.001)	3.03	1.31–7.02	0.010
Urethral discharge, lifetime				
No	Ref	Ref	-	-
Yes	3.20 (<0.001)	1.64	0.73–3.67	0.230
History of an STI, lifetime				
No	Ref	Ref	-	-
Yes	4.92 (<0.001)	2.44	1.08–5.49	0.032

* This variable was entered as the log base 10 of the reported number of lifetime partners.

** All odds ratios are adjusted for all of the other variables in the model as well as age.

## Discussion

In our survey of men living in low-income neighborhoods in urban Peru, we found that men who reported engaging in same-sex sexual behavior during their lifetime were more likely than men who claimed exclusively female sexual contacts to engage in high-risk sexual behavior, to have had an STI in the past, and to be HSV-2 seropositive. Our findings are consistent with a variety of studies conducted in diverse international settings [1], [4], [5], [12], [21]–[24], [33]–[40], but should be interpreted within Peru's specific cultural context.

In our study, MESM had a greater number of lifetime sexual partners, and were less likely to report using condoms with their casual sexual partners, suggesting that they had more frequent, often unprotected, casual sexual contacts than non-MESM. Research conducted in low-income communities in Peru and other Latin American societies has suggested that the cultural segregation of sexual roles into masculine, heterosexual-identified *activos* and feminized, homosexual-identified *pasivos* enables heterosexual-identified men to engage in same-sex encounters without significantly jeopardizing their masculine social status [13], [14], [23]. However, this system of gendered sexual identities typically limits homosexual men to transient sexual encounters with heterosexual-identified men who routinely reject stable same-sex relationships [14], [18], [26].

In addition to these casual sexual contacts, more than one-third (36.5%) of men who reported previous sex with men also reported receiving money or goods in exchange for sex during their lifetime. Participation in compensated sex results in greater exposure to STIs and more frequent high-risk behavior with clients who will pay more money for unprotected intercourse [16], [18]. In the setting of compensated sex, male sexual contact is redefined as a commercial transaction where self-protective behavior is designated as a factor in a financial negotiation, rather than an intimate agreement between sexual partners.

We also observed a similar frequency of reported MESM activity between 18 year olds (15.9%) and 19–30 year olds (15.2%), suggesting that same-sex sexual contact, either as an isolated experiment or as a regular practice, is often initiated during adolescence. Initial sexual experiences with homosexual-identified men are frequently accepted in low-income Peruvian communities without necessarily identifying the adolescent as gay or bisexual [11], [23], [26]. Through this system of sexual initiation, “homosexual” men are used as sexual objects by young men in the community to gain experience before forming stable, normative heterosexual partnerships [11], [14]. In addition to marginalizing gay-identified MSM, this pattern of socialization places adolescent males in intimate contact with a high-risk group for HIV and STIs at a time in which they are least likely to possess adequate knowledge concerning prevention.

Though we did not identify any cases of HIV in our study sample, multiple elements conducive to the spread of HIV infection among MESM were present. Increased prevalences of HSV-2 and syphilis, greater rates of prior STI diagnoses and symptoms, as well as more lifetime partners and more frequent unprotected sex contacts were observed in MESM compared to non-MESM. Unfortunately, while almost one-third of the MESM in our study rated themselves as moderate or high risk for HIV infection, they were no more likely to have been tested for HIV or to have tried to obtain condoms. Though currently untouched by HIV, the MESM in our study provide a behavioral and biological context conducive to the extensive spread of infection following the introduction of HIV into their networks.

There are several limitations to our study, primarily centered on the fact that the survey was designed to evaluate risk behaviors and disease prevalence in a general population sample, without specifically assessing MSM. Participants were not asked whether they identified as heterosexual, homosexual, or bisexual, and the gender of individual sexual contacts was not specified. As a result, MESM are collapsed into a single analytic category that erases social and behavioral differences between subpopulations which may be strongly associated with risk for HIV and STIs. Participants were also not provided with a definition of the term “sex,” an omission that introduces further ambiguity into our findings by requiring participants to supply their own, inconsistent, meanings for the term. Due to stigma surrounding homosexuality and participant underreporting of same-sex activity, we have likely underestimated the prevalence of this activity. In addition, it is difficult to assess whether the higher prevalence of risk behavior reported by MESM is directly related to their sexual contact with other men or if there is a correlation between previous male same-sex sexual contact and a nonspecific propensity to engage in high-risk sexual behavior. The predominance of women in our sample reflects a potential bias in our household-based recruitment strategy since Peruvian men are traditionally expected to be engaged in work or (potentially high-risk) leisure activities outside the home and are less likely to be at home during recruitment visits. However, the household design of our study provides a more representative sample than analyses based on convenience or snowball sampling from MSM-associated venues, and provides the most accurate available measure of MESM risk behavior in Peru. Though Respondent-Driven Sampling (RDS) [41]–[44] and Time-Location Sampling (TLS) [45] may be valuable in future studies for identifying representative samples of MSM in Peru, household sampling is the only methodology known to us that can obtain a random sample that includes men whose sexual contacts with men were entirely in the past and therefore can provide an estimate of all men who have *ever* had sex with men (MESM). Despite their limitations, our results indicate that MESM are at higher risk for STI transmission and potentially for HIV acquisition than men who denied any lifetime sexual contact with men.

Men who reported ever having “sex” with other men in low-income urban communities in Peru were more likely to engage in high-risk sexual behavior, and had a higher prevalence of STIs than men who did not describe previous same-sex contact. HIV and STI prevention programs need to be developed that are directed to the general population and emphasize the increased risk faced by all men who have sex with men, regardless of their sexual identity, and address behavioral change within the specific contexts of male sexuality in Peruvian society. Continued study of the contexts of male sexual contact in Latin America and their relation to sexual identity and risk behavior is essential for accurate epidemiologic surveillance and improved strategies for the prevention of HIV and STIs.
